# Supplementary material for: Evolution of masting in plants is linked to investment in low tissue mortality
Source: Nat Commun. 2023 Dec 2;14:7998. doi: 10.1038/s41467-023-43616-1 (PMC10693562; doi:10.1038/s41467-023-43616-1)
Supplement: Supplementary file 1 — Supplementary Information [file 41467_2023_43616_MOESM1_ESM.pdf]

Supplementary material

Supplementary Notes

Supplementary Note 1: Analysis with time-series restricted to 10 years

In that Supplement, we report the results as in the main text, but with a more restrictive data filtering, i.e. we limited the time series (site by species combinations) to have at least 10 years of observations.

Supplementary Note 2: Phylogenetic signal The strength of the phylogenetic signal slightly increased once the data was restricted to fewer species. In the case of CV, λ equaled 0.57 ($p < 0.00001$, n = 364 species), while in the case of AR1, λ equaled 0.41 ($p < 0.00001$, n = 364 species, Fig. S10).

Supplementary Note 3: Principal Component Analysis (PCA) Patterns summarized by the PCA analysis on the restricted dataset (n = 368) resembled those run on a larger set of species (n = 517). Masting metrics created a 3rd, largely independent from the first two, axis of variation (Fig. S11).

Supplementary Note 4: Generalized Joint Attribute Modeling (GJAM) The coefficient of variation and lag-1 temporal auto-correlation responded in the opposite way to climate (Fig. S12). Conditional parameters estimated with GJAM support the conclusion that high CV is concentrated in species that are characterized by conservative tissue construction, i.e. high stem (tissue) density (Fig. S13). That support comes from the relationship of CV with stem tissue density, but not with leaf mass per area (LMA).

Supplementary Figures

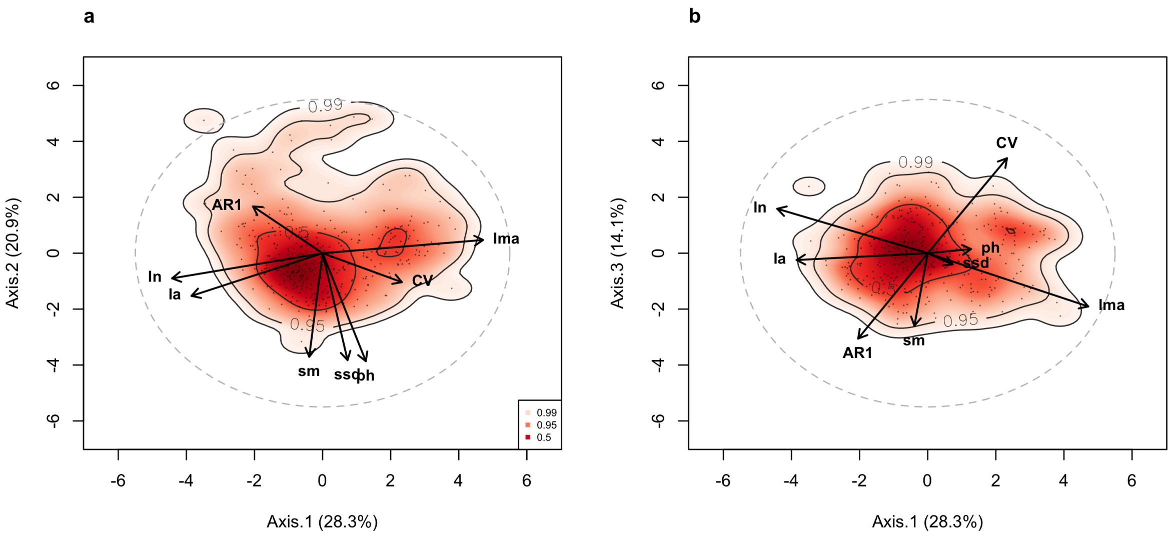

Figure S1: **Masting metrics on the spectrum of plant form and function.** Trait probability density function for principal components: a) axis 1 and axis 2; b) axis 1 and axis 3. The red color gradient indicates the probabilistic distribution of trait combinations in the functional trait space defined by a PCA. Contour lines indicate 0.99, 0. 95, and 0.50 quantiles of the probability distribution, and dots represent species. We estimated the occurrence probability of a given combination of trait values determined by the principal components axis and bivariate trait combination using two-dimensional kernel density estimation.

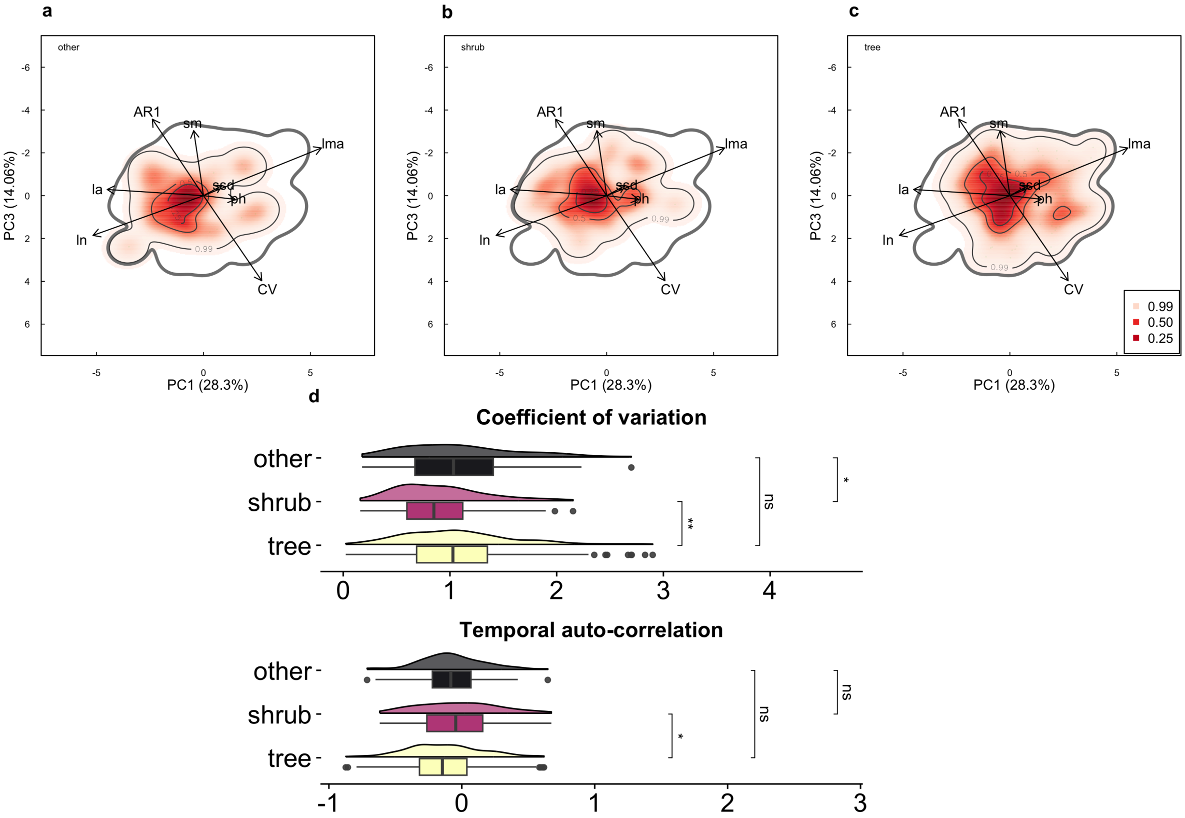

Figure S2: **Masting metrics on the spectrum of plant form and function, by growth form.** Trait probability density function for principal components between axis 3 and axis 1 according to plant growth form for a) other groups, b) shrub, and c) trees. For each growth form group, the colors indicate the probabilistic distribution of trait combinations in the functional trait space defined by a PCA (ranging from low probability in pale white to high probability in red). Contour lines indicate 0.99, 0. 50, and 0.25 quantiles of the probability distribution. We estimated the occurrence probability of a given combination of trait values determined by the principal components axis and bivariate trait combination using two-dimensional kernel density estimation. Analysis and plots have been made with the R package `funspace` [51]. d) Coefficient of variation (CV) and lag-1 temporal auto-correlation (AR1) across growth forms (n = 517 species). The growth form follows a compilation from [49], with samples: trees, n = 367 species; shrubs, n = 86 species; other n = 64 species. Other include graminoid and non-graminoid herbaceous and climbers. Groups were compared with a one-sample t-test (** $P <$ for 0.01, * $P <$ for 0.05, and n.s. for $P > 0.05$)

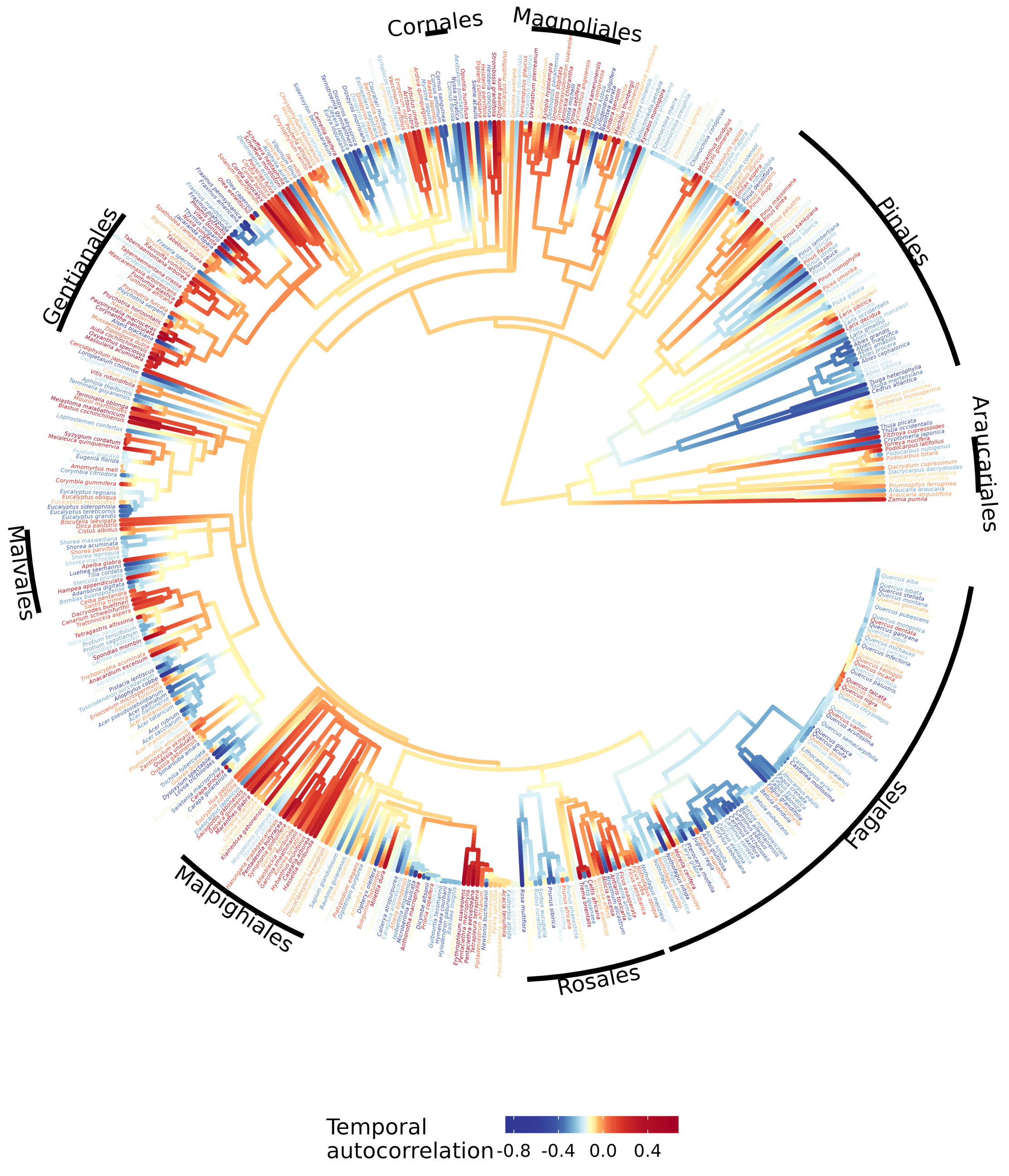

Figure S3: **Lag-1 temporal autocorrelation of seed production mapped onto a plant phylogeny.** Warmer colors (reds) indicate higher, while blue lower AR1 ($\lambda = 0.27$, $p < 0.0001$, n = 518 species). Distribution of the masting metrics is given in Fig. S4.

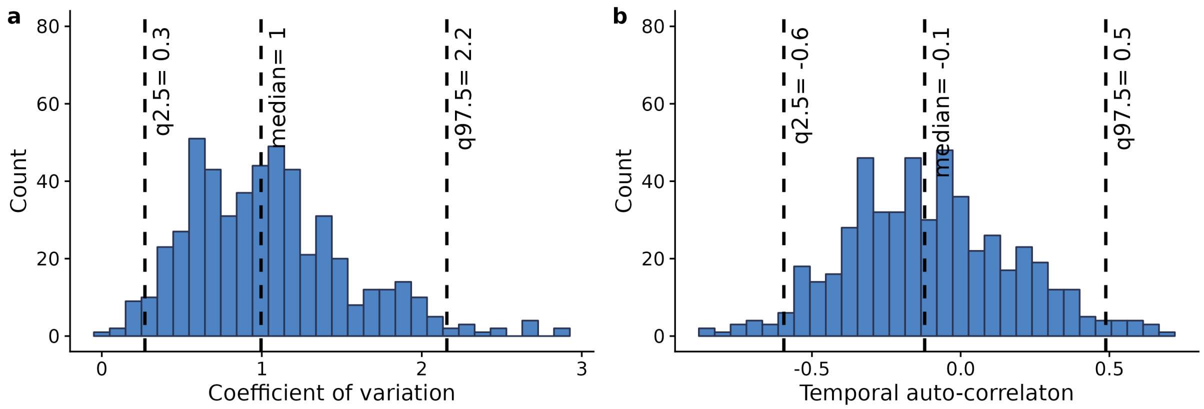

Figure S4: **Distribution of masting metrics.** Histogram of a) coefficient of variation (CV), and b) lag-1 temporal auto-correlation (AR1) for the 517 species analyzed. Black dotted lines show median and quantiles at 2.5% and 97.5%.

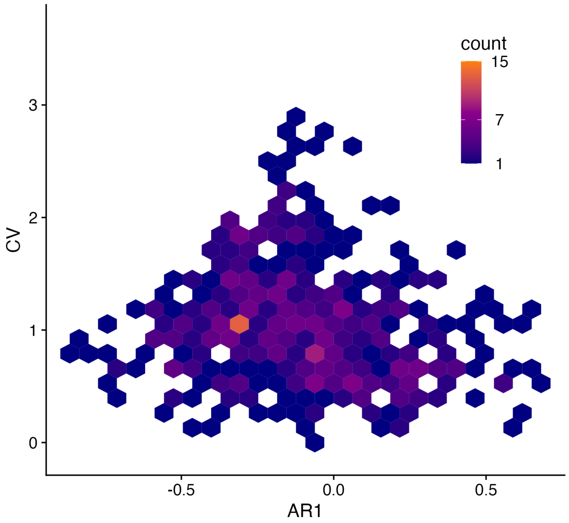

Figure S5: **Relationship between the coefficient of variation (CV) and the lag-1 temporal auto-correlation (AR1)**. The Hexagon color is scaled to the number of observations within each hexagon, n = 517.

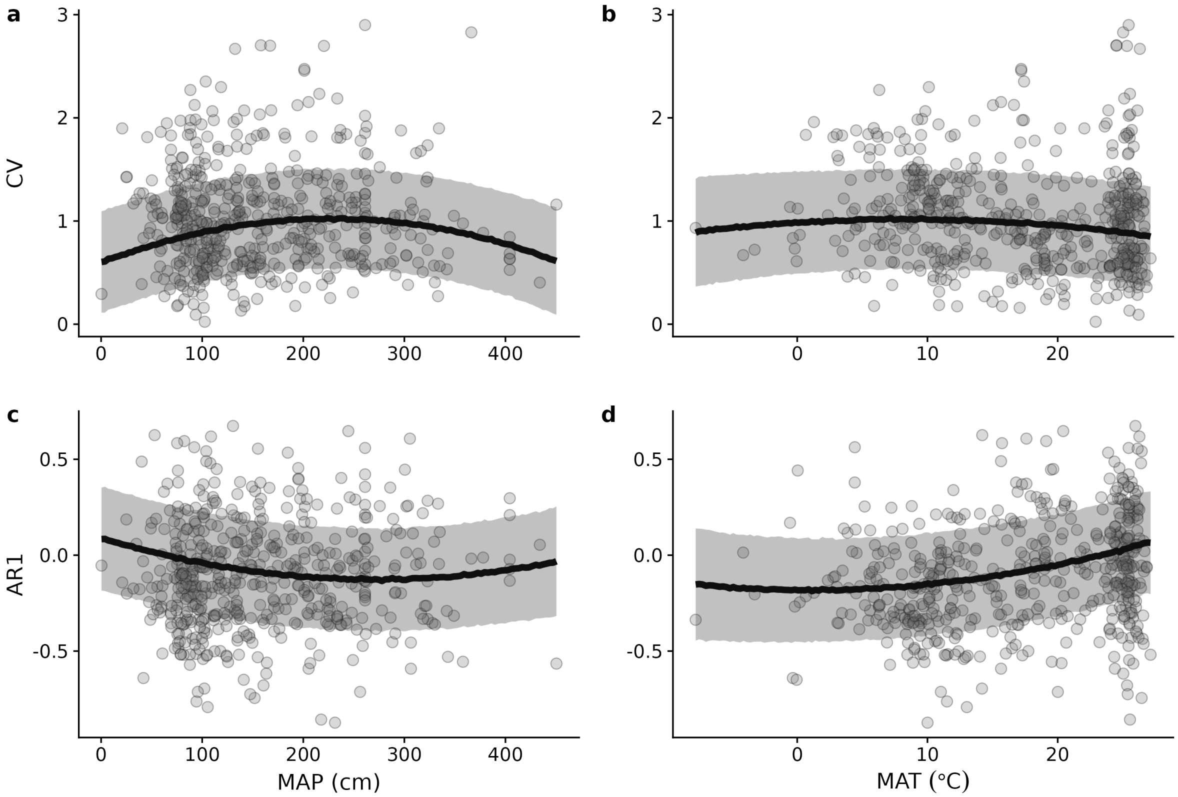

Figure S6: **Summary of climate effect on masting metrics, derived from the GJAM model.** Relationship between the coefficient of variation (CV) and species climatic niche: a) MAP, in cm, and b) MAT, in °C. Relationship between lag-1 temporal auto-correlation (AR1) and species climatic niche: c) MAP, in cm, and d) MAT in °C). The predictions and associated standard error are derived from the GJAM model. Each dot represents one species (n = 517).

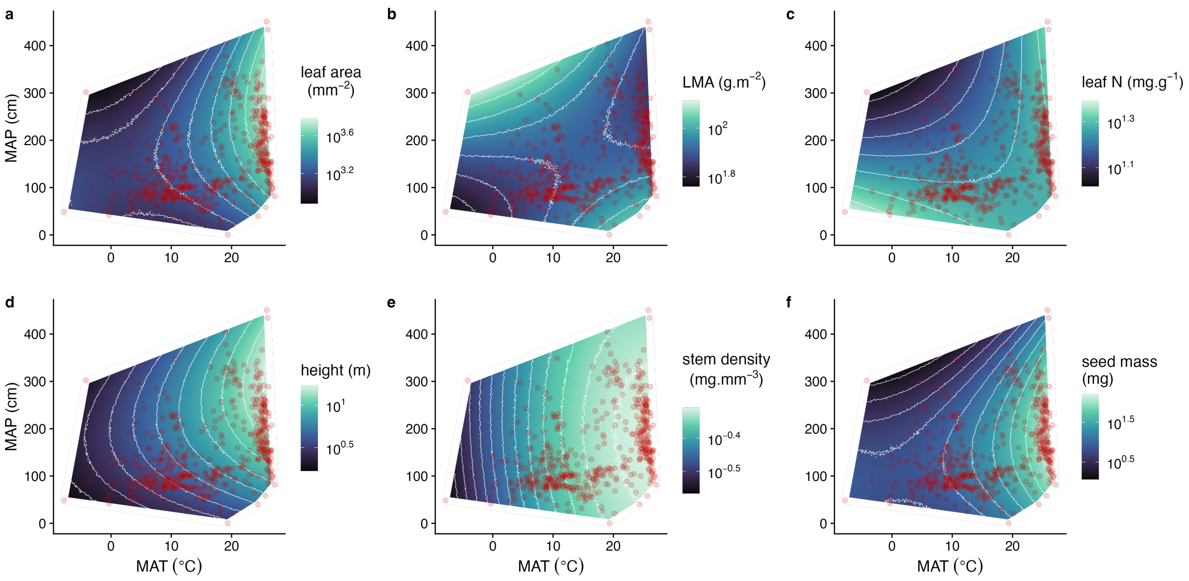

Figure S7: **Summary of climate effect on functional traits, derived from the GJAM model.** Effects of mean annual temperature (MAP, in $°C$) and mean annual precipitation (MAT, in cm) on functional traits (a- leaf area, b- LMA, c- leaf N, d- plant height, e- stem tissue density and f- seed mass). The surface shows the conditional relationship between functional traits and MAT across levels of MAP. Convex hull is defined by species observations (red circles). MAT and MAP are defined for each species' distribution derived from Global Biodiversity Information Facility (GBIF, www.gbif.org). Traits are log10 transformed.

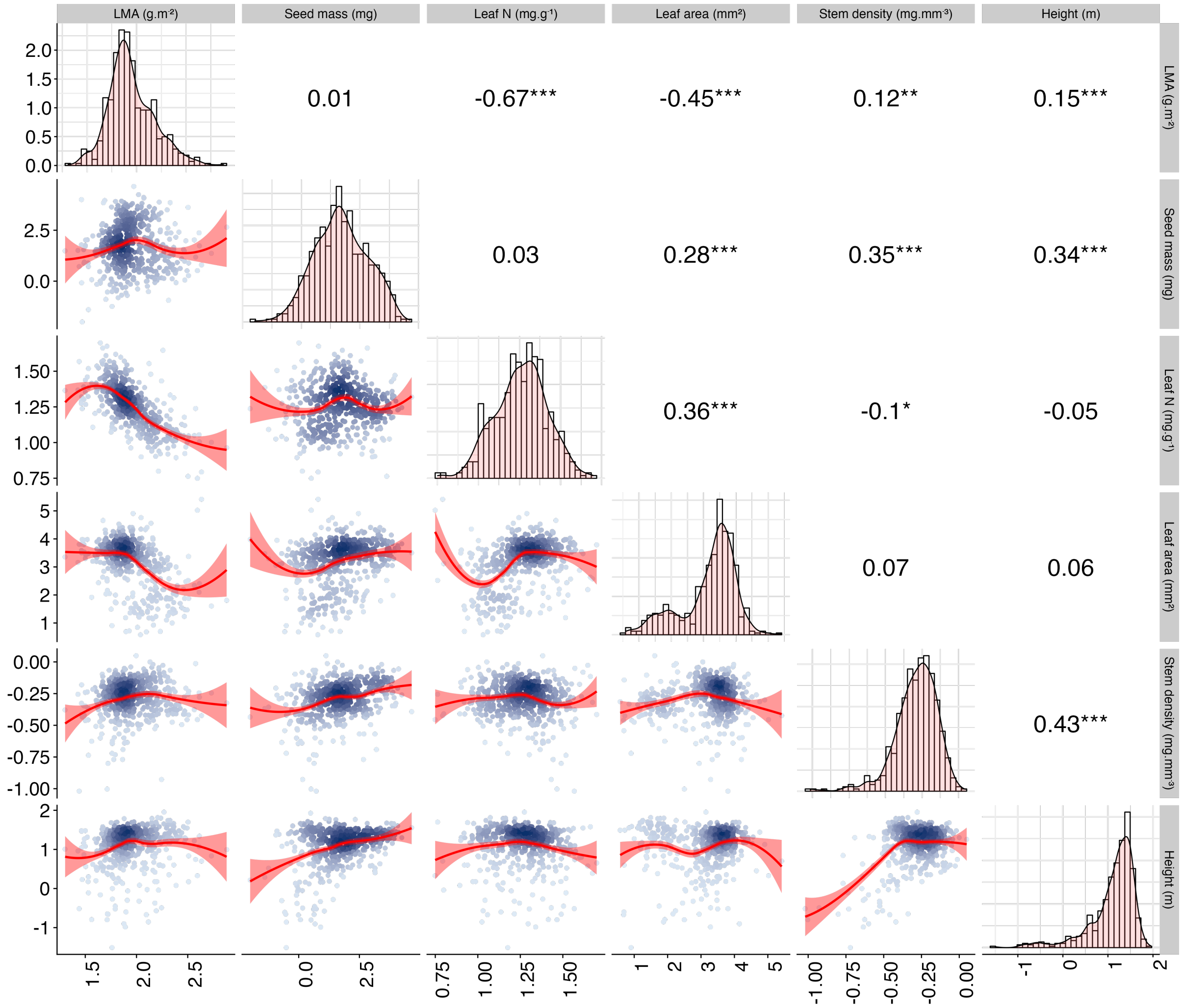

Figure S8: **Bivariate relationships between plant functional traits, their distributions (histograms), and correlations.** The prediction lines, given in red, at bivariate scatter plots are the loess regressions (estimate with 95% CI). The blue dots represented each species (n=517), with 2D kernel density estimation. The significance of the Pearson correlations: *** for p-values < 0.001, ** for 0.001, and * for 0.01. Traits were log10 transformed.

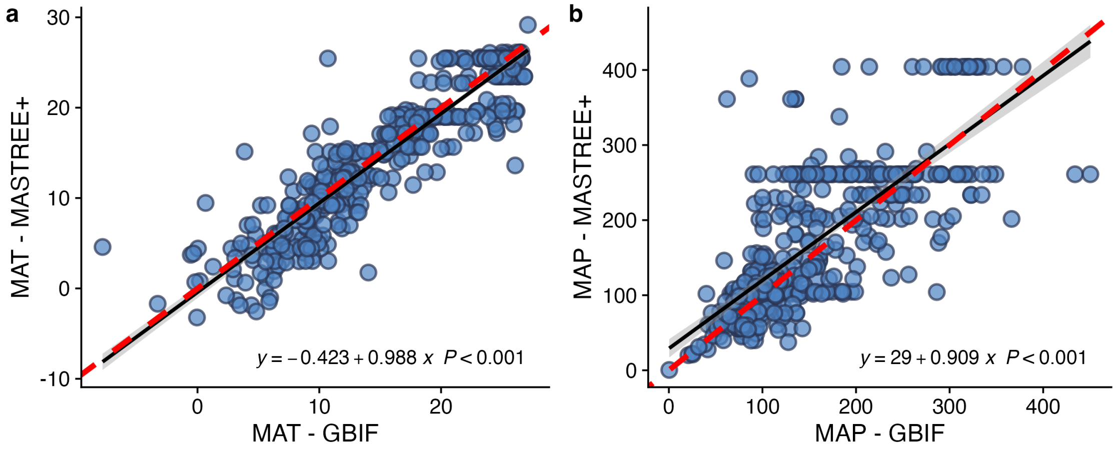

Figure S9: **Correlation of climatic variables obtained from average conditions of MASTREE+ sites and from GBIF observations.** a) Relation between MAT (in degree C) from MASTREE+ observations and GBIF observations extracted from CHELSA. b) Relation between MAP (in cm) from MASTREE+ observations and GBIF observations extracted from CHELSA. Each dot represents one species (n = 517). The regression line is reported in black (estimate with 95 % CI), with the equation at the bottom right and correlation and the 1:1 line in red dashed.

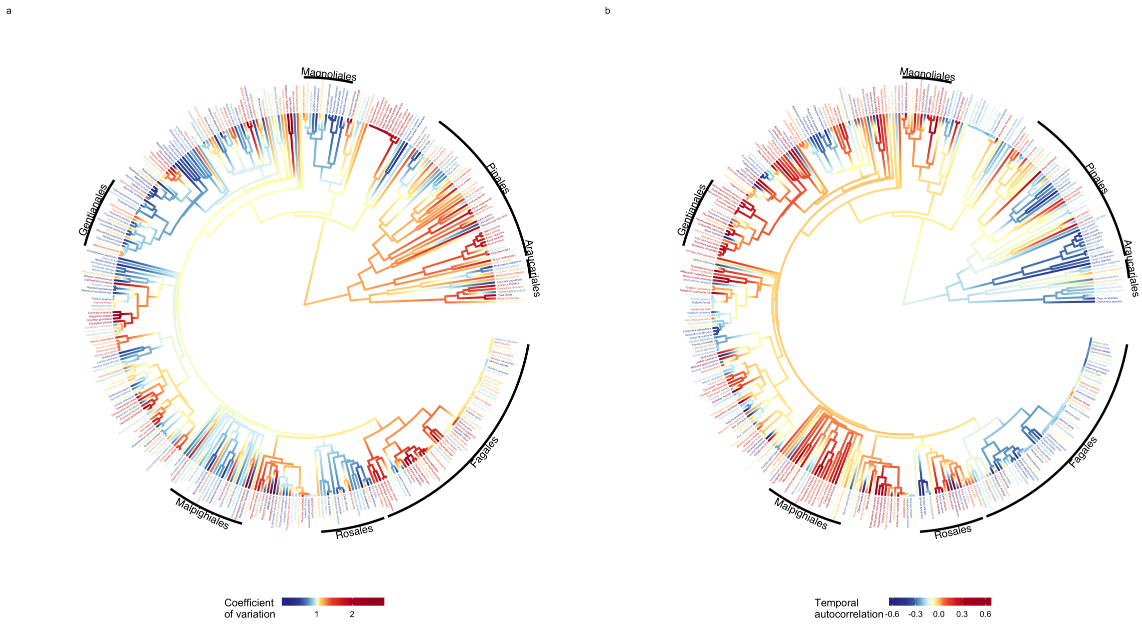

Figure S10: **Phylogeny of masting metrics on a restricted dataset.** (a) Coefficient of variation of seed production mapped onto a plant phylogeny restricted to time series of 10 years and longer. Warmer colors (reds) indicate higher, while blue lower CV (λ = 0.56, p < 0.0001, n = 364 species). (b) Lag-1 temporal autocorrelation of seed production mapped onto a plant phylogeny, restricted to time series of 10 years and longer. Warmer colors (reds) indicate higher, while blue lower temporal autocorrelation (λ = 0.40, p < 0.0001, n = 364 species).

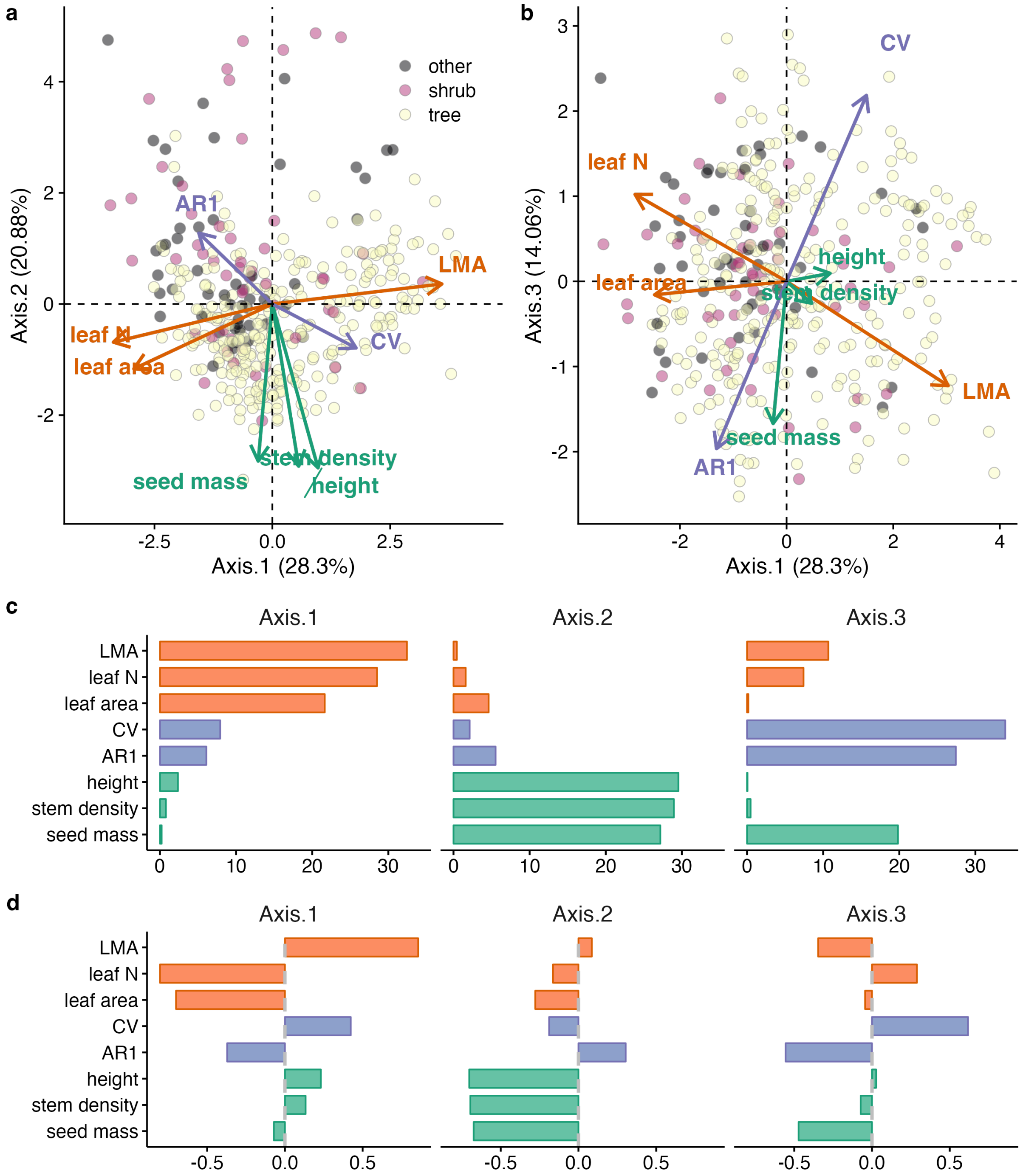

Figure S11: **Masting metrics on the spectrum of plant form and function**, for time series of 10 years and longer (n=368). A) Biplot of principal components that summarized axes 1 and 2, and B) axes 1 and 3. The PCA included plant functional traits (stem tissue density, leaf area, leaf nitrogen, leaf mass per area LMA, plant height, and seed mass) and masting metrics (CV and AR1). Arrow length indicates the loading of each considered trait onto the axes. Points represent the position of species color-coded according to their growth form (green for trees, blue for shrubs, and black for others that included graminoid and non-graminoid herbaceous and climbers). C) Summary of PCA loadings and D) contributions to the three axes of variation.

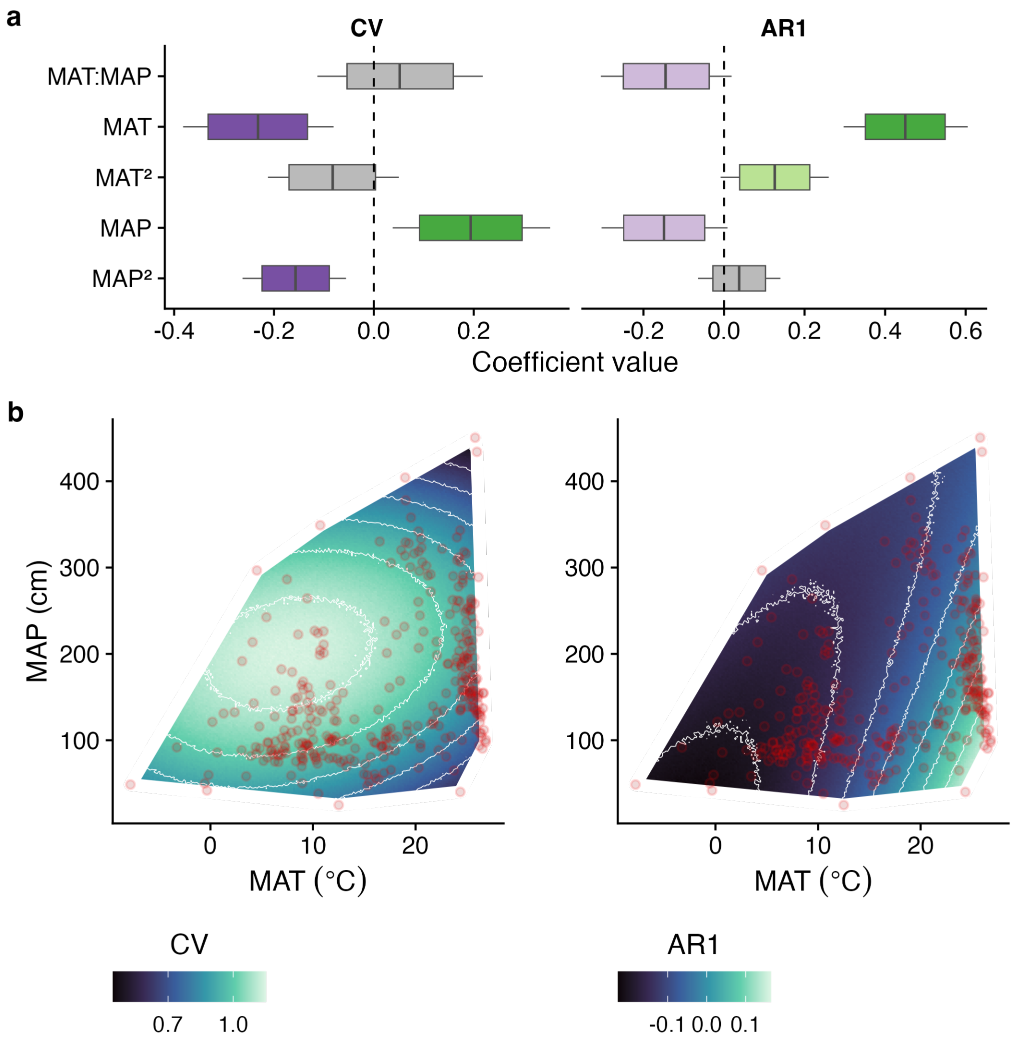

Figure S12: **Summary of climate effects on masting metrics**, derived from the GJAM model that included coefficient of variation (CV) and temporal autocorrelation (AR1) as responses for time series of 10 years and longer (n=368 species). a) Boxplot of standardized coefficients from the GJAM model with 95%CI, bounded by 80% interval. Colors highlight signs of the correlation (green for positive and purple for negative), with opacity increasing from 80% to 95% of the distribution outside of zero. Grey is for coefficients that overlap zero. b) Effects of mean annual temperature (MAP, in $°C$) and mean annual precipitation (MAT, in cm) on CV and AR1. The surface shows the conditional relationship between CV/AR1 and MAT across levels of MAP. Convex hull is defined by species observations (red dots). MAT and MAP are defined for each species' distribution derived from the Global Biodiversity Information Facility (GBIF, www.gbif.org). Biplots of relationships between CV/AR1 and MAT and MAP are in Fig. S6.

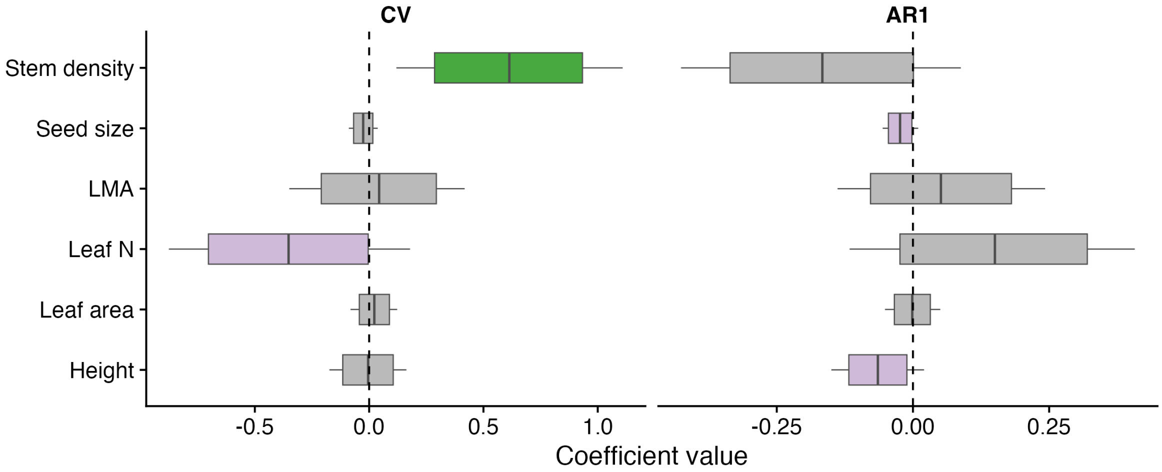

Figure S13: **Conditional relationship between masting metrics and functional traits**, restricted to time series of 10 years and longer (n=368 species). Boxplots are based on the mean estimate, CI at 80% and 95% to determine the ranges of the boxplot. Colors highlight signs of the correlation (green for positive and purple for negative), with opacity increasing from 80% to 95% of the distribution outside of zero. Grey is for non-significant variables (i.e. coefficients overlap 0).

Supplementary Tables

Table S1: **Summary of the conditional relationship between masting metrics and functional traits without trait imputation**. GJAM-derived conditional relationship between masting metrics (CV and AR1) and functional traits (stem tissue density, seed size, LMA, leaf N, leaf area, and plant height) after accounting for the effect of climate and phylogeny. Coefficients are reported with 95%CI, with significance (95% CI overlapping 0) of functional trait coefficients in bold. GJAM was used here on the dataset without functional trait imputation (total count of species with missing traits for LMA = 90 species; seed size = 84 species; leaf N = 96 species; leaf area = 111 species; stem density = 84 species; height = 51 species).

Masting metric	Conditional traits	Estimate	SE	2.5%	97.5%	significance
**CV**						
	**LMA**	9.64e-04	4.26e-04	1.38e-04	1.79e-03	*
	**Seed size**	-2.27e-05	8.60e-06	-3.92e-05	-5.60e-06	*
	Leaf N	-6.98e-04	3.95e-03	-8.44e-03	7.18e-03	
	Leaf area	-8.00e-07	1.50e-06	-3.80e-06	2.10e-06	
	**Stem density**	5.68e-01	1.79e-01	2.17e-01	9.29e-01	*
	Height	3.97e-03	2.17e-03	-2.33e-04	8.28e-03	
**AR1**						
	LMA	-3.17e-05	2.36e-04	-4.94e-04	4.34e-04	
	Seed size	4.40e-06	4.80e-06	-4.80e-06	1.38e-05	
	**Leaf N**	4.82e-03	2.35e-03	3.11e-04	9.45e-03	*
	Leaf area	2.00e-07	8.00e-07	-1.50e-06	1.90e-06	
	Stem density	-1.01e-01	9.88e-02	-2.92e-01	9.72e-02	
	**Height**	-2.77e-03	1.20e-03	-5.08e-03	-4.06e-04	*

Table S2: **Joint traits model selection (based on the DIC values)**. GJAM models were fitted with different combinations of climate covariates, average species climatic conditions (MAP and MAT), and climate variability (MAP_σ and MAT_σ). Some model combinations were excluded due to collinearity issues. Note: in the top-scored models that included climate variability, the effects of climate variability on masting metrics overlapped with 0. The other models that included either MAP_σ and MAT_σ had the Δ DIC less than 10, which means that these model fits received essentially no support. In other words, the probability that one of the alternative models is the best for the data is 0.

Climatic predictors in GJAM	DIC
$MAP \times MAT + MAT^2 + MAP^2$	10,997
$MAP \times MAT + MAT^2 + MAP^2 + MAP_\sigma$	11,005
$MAP \times MAT$	11,063
$MAP_\sigma \times MAT + MAT^2 + MAP_\sigma^2$	11,113
$MAP_\sigma \times MAT$	11,149
$MAT_\sigma \times MAT$	11,159
$MAP \times MAT_\sigma + MAT_\sigma^2 + MAP^2 + MAP_\sigma$	11,190
$MAP_\sigma \times MAT_\sigma + MAT_\sigma^2 + MAP_\sigma^2$	11,314
$MAP_\sigma \times MAP + MAP^2 + MAP_\sigma^2$	11,464
$MAP \times MAT_\sigma$	11,505
$MAP \times MAP_\sigma$	11,576
$MAP_\sigma \times MAT_\sigma$	11,652
