# Supplementary material for: Evolution of masting in plants is linked to investment in low tissue mortality
Source: Nat Commun. 2023 Dec 2;14:7998. doi: 10.1038/s41467-023-43616-1 (PMC10693562; doi:10.1038/s41467-023-43616-1)
Supplement: Supplementary file 3 — Reporting Summary [file 41467_2023_43616_MOESM3_ESM.pdf]

nature portfolio

Corresponding author(s): Valentin Journé

Last updated by author(s): Oct 23, 2023

Reporting Summary

Nature Portfolio wishes to improve the reproducibility of the work that we publish. This form provides structure for consistency and transparency in reporting. For further information on Nature Portfolio policies, see our Editorial Policies and the Editorial Policy Checklist.

Statistics

For all statistical analyses, confirm that the following items are present in the figure legend, table legend, main text, or Methods section.

n/a	Confirmed	
☐	☒	The exact sample size (*n*) for each experimental group/condition, given as a discrete number and unit of measurement
☒	☐	A statement on whether measurements were taken from distinct samples or whether the same sample was measured repeatedly
☐	☒	The statistical test(s) used AND whether they are one- or two-sided<br>*Only common tests should be described solely by name; describe more complex techniques in the Methods section.*
☐	☒	A description of all covariates tested
☐	☒	A description of any assumptions or corrections, such as tests of normality and adjustment for multiple comparisons
☐	☒	A full description of the statistical parameters including central tendency (e.g. means) or other basic estimates (e.g. regression coefficient) AND variation (e.g. standard deviation) or associated estimates of uncertainty (e.g. confidence intervals)
☐	☒	For null hypothesis testing, the test statistic (e.g. *F*, *t*, *r*) with confidence intervals, effect sizes, degrees of freedom and *P* value noted<br>*Give P values as exact values whenever suitable.*
☐	☒	For Bayesian analysis, information on the choice of priors and Markov chain Monte Carlo settings
☐	☒	For hierarchical and complex designs, identification of the appropriate level for tests and full reporting of outcomes
☐	☒	Estimates of effect sizes (e.g. Cohen's *d*, Pearson's *r*), indicating how they were calculated

Our web collection on statistics for biologists contains articles on many of the points above.

Software and code

Policy information about availability of computer code

Data collection	No software have been used for data collection.
Data analysis	All analysis have been done through the R software (v4.3.0). All analyses used published R packages.

For manuscripts utilizing custom algorithms or software that are central to the research but not yet described in published literature, software must be made available to editors and reviewers. We strongly encourage code deposition in a community repository (e.g. GitHub). See the Nature Portfolio guidelines for submitting code & software for further information.

Data

Policy information about availability of data

All manuscripts must include a data availability statement. This statement should provide the following information, where applicable:

- Accession codes, unique identifiers, or web links for publicly available datasets
- A description of any restrictions on data availability
- For clinical datasets or third party data, please ensure that the statement adheres to our policy

Our study is based on a MASTREE+ dataset that is is available from https://github.com/JJFoest/MASTREEplus with details provided in Hacket-Pain et al. Traits have been downloaded from the recent compilation of Diaz et al. Species occurences have been obtained through GBIF (www.gbif.org). Climate data have been extracted from CHELSA at https://chelsa-climate.org/.

April 2023

Research involving human participants, their data, or biological material

Policy information about studies with human participants or human data. See also policy information about sex, gender (identity/presentation), and sexual orientation and race, ethnicity and racism.

Reporting on sex and gender	N/A
Reporting on race, ethnicity, or other socially relevant groupings	N/A
Population characteristics	N/A
Recruitment	N/A
Ethics oversight	N/A

Note that full information on the approval of the study protocol must also be provided in the manuscript.

Field-specific reporting

Please select the one below that is the best fit for your research. If you are not sure, read the appropriate sections before making your selection.

☐ Life sciences ☐ Behavioural & social sciences ☒ Ecological, evolutionary & environmental sciences

For a reference copy of the document with all sections, see nature.com/documents/nr-reporting-summary-flat.pdf

Ecological, evolutionary & environmental sciences study design

All studies must disclose on these points even when the disclosure is negative.

Study description	We investigate interannual patterns of reproductive investment in relation to functional traits, climate, and phylogeny in 517 species of perennials, including herbs, graminoids, shrubs, and trees.
Research sample	Each observation is a an annual, population-level seed production quantified at the site-species level. It can be a number of seed per trap, a number of seed in a tree, or a percentage over the canopy. We determined for each site and species the coefficient of variation and the lag1 temporal autocorrelation and averaged them for each species.
Sampling strategy	The study is based on a MASTREE+ dataset that compiles population-level observations of annual seed production in perennial plants. MASTREE+ data was extracted from published sources.
Data collection	As above, i.e. The study is based on a MASTREE+ dataset that compiles population-level observations of annual seed production in perennial plants. MASTREE+ data was extracted from published sources.
Timing and spatial scale	The mean and median time-series length is 12.4 and 10 years respectively, and the data set includes 1122 series that extend over at least two decades (≥20 years of observations). These observations consist of 5971 population-level time-series from 974 species in 66 countries, from all vegetated continents.
Data exclusions	From MASTREE+ dataset, we excluded time series that were on an ordinal scale and those based on pollen measurements. Justification is given in the main text. We conducted analysis limited to time series with at least 5 years of observations and 10 years of records.
Reproducibility	All analysis are based on published and available R packages on an open-source datasets.
Randomization	This is an observational study.
Blinding	This is an observational study.

Did the study involve field work? ☐ Yes ☒ No

Reporting for specific materials, systems and methods

We require information from authors about some types of materials, experimental systems and methods used in many studies. Here, indicate whether each material, system or method listed is relevant to your study. If you are not sure if a list item applies to your research, read the appropriate section before selecting a response.

Materials & experimental systems

n/a	Involved in the study
☒	☐ Antibodies
☒	☐ Eukaryotic cell lines
☒	☐ Palaeontology and archaeology
☒	☐ Animals and other organisms
☒	☐ Clinical data
☒	☐ Dual use research of concern
☒	☐ Plants

Methods

n/a	Involved in the study
☒	☐ ChIP-seq
☒	☐ Flow cytometry
☒	☐ MRI-based neuroimaging
